# Reply

**DOI:** 10.1055/s-0044-1786025

**Published:** 2024-04-12

**Authors:** Leonardo Furtado Freitas, Eduardo Carvalho Miranda, Thelma Ribeiro Noce, Aline Pimentel Amaro, Márcio Luís Duarte

**Affiliations:** 1 McGill University, Department of Radiology, Division of Neuroradiology, Montreal QC, Canada.; 2 Neurodigital, São Paulo SP, Brazil.; 3 Rede MaterDei de Saúde, Departamento de Neurorradiologia, Belo Horizonte MG, Brazil.; 4 Rede MaterDei de Saúde, Departamento de Neurologia Infantil, Belo Horizonte MG, Brazil.; 5 Hospital Felício Rocho, Belo Horizonte MG, Brazil.; 6 Universidade de Ribeirão Preto, Guarujá SP, Brazil.

The pathogenicity of variant c.597dup in *SLC19A3* and treatability of its phenotype remain unconfirmed

Dear Editor,

Initially, we would like to thank Josef Finsterer and Fulvio Scorza for their comments and for the opportunity to discuss this case report. 1 There is a typing error, and the correct name of the gene in question is SLC19A3, whose inheritance is indeed autosomal recessive.

Genetic exome analysis showed 2 different mutations in the SLC19A3 gene. A pathogenic variant c.597dup p.(His200Serfs*25) and a variant of uncertain significance (VUS) c.488C > T p.(Ser163Phe). These mutations are on different alleles (compound heterozygosity), which would function as homozygosity. This would explain the disease and the variant of uncertain significance should be reclassified as probably pathogenic. So far, therefore, we do not doubt the diagnosis, after a multidisciplinary discussion with a pediatric neurologist and geneticist.

The patient in question did not die, and showed a slight clinical improvement with treatment, despite having sequelae and a very poor prognosis due to the parenchymal changes in the brain, even with high-dose treatment. He is currently taking 500 mg of thiamine and 80 mg of biotin in the morning and 300 mg of thiamine and 40 mg of biotin in the evening.

The parents' genetic tests have been requested but are not yet available at the time of this letter.

We would like to thank you again for the comments and the possibility of corrections and clarifications.
